# Herniation of the Urinary Bladder Into the Scrotal Sac With Intrascrotal Perforation: A Report of a Rare Case

**DOI:** 10.7759/cureus.69574

**Published:** 2024-09-17

**Authors:** Arun Aram, Vadupu Udaya Bhanu, Arunkumar Mohanakrishnan, Paarthipan Natarajan

**Affiliations:** 1 Department of Radiology, Saveetha Medical College and Hospital, Saveetha Institute of Medical and Technical Sciences (SIMATS) Saveetha University, Chennai, IND

**Keywords:** bladder herniation, bladder perforation, inguinal hernia, intrascrotal perforation, scrotal cystocele

## Abstract

This case report describes a rare and complex instance of urinary bladder herniation into the scrotal sac, complicated by intrascrotal perforation. This condition, primarily seen in elderly, obese men, poses significant diagnostic and therapeutic challenges. The patient, an 83-year-old man with a history of obesity and chronic lower urinary tract symptoms, presented with acute scrotal pain and swelling. Physical examination revealed a tender, enlarged scrotum with a palpable mass. Imaging studies, including ultrasound and computed tomography, confirmed bladder herniation into the scrotal sac with evidence of perforation. Prompt surgical intervention involved partial cystectomy to remove the perforated segment of the bladder and repair the hernia. The procedure was successful, and the patient experienced an uneventful postoperative recovery with no recurrence or complications. Follow-up assessments showed a return to normal urinary function. This case underscores the critical importance of timely diagnosis and intervention in managing such rare and potentially life-threatening conditions, particularly in elderly, obese males presenting with scrotal swelling and pain.

## Introduction

Urinary bladder herniation through the inguinal canal into the scrotum, known as scrotal cystocele, represents a rare but clinically significant phenomenon that occurs in approximately 1-4% of all inguinal hernias. While inguinal hernias themselves are a relatively common condition, scrotal cystocele specifically involves the herniation of the bladder and is often associated with unique clinical challenges. This condition is predominantly seen in elderly, obese males who are predisposed to anatomical and physiological changes that increase their vulnerability [1].

The pathogenesis of scrotal cystocele can be attributed to a combination of factors such as weakened abdominal wall musculature, increased intra-abdominal pressure, and age-related degeneration of connective tissues. These elements, often present in elderly or overweight individuals, collectively facilitate the abnormal protrusion of the bladder into the scrotum. Several factors increase the likelihood of developing a scrotal cystocele, including chronic straining due to constipation, chronic cough (often associated with smoking), or heavy lifting, all of which lead to increased intra-abdominal pressure [2]. Additionally, patients with a history of multiple inguinal hernias, previous hernia repairs, or pelvic surgery are at increased risk, as prior operations may further weaken the anatomical barriers that prevent herniation. These anatomical and physiological alterations, when combined, allow the bladder to gradually descend into the inguinal canal and scrotal sac over time, ultimately leading to the formation of a scrotal cystocele [1,2].

Clinically, scrotal cystocele often presents with lower urinary tract symptoms, such as dysuria (painful urination), increased urinary frequency, urgency, and occasionally hematuria (blood in the urine). These symptoms arise due to the mechanical effects of the herniated bladder, which may obstruct or irritate the urinary tract. Additionally, patients may notice a swelling or bulge in the groin or scrotum, which may fluctuate in size depending on bladder filling and voiding [2]. Some individuals may even report the need to manually compress or “milk” the scrotal swelling to aid in bladder emptying, a phenomenon known as manual reduction or “voiding by pressure.”

The pathophysiology underlying scrotal cystocele involves the migration of the bladder through a weakened inguinal canal into the scrotum [3]. This process is exacerbated by elevated intra-abdominal pressures and a diminished structural support system, including weakened fascia and connective tissue. As the bladder herniates into the scrotum, it may become compressed, resulting in urinary outflow obstruction and the aforementioned symptoms. Although rare, bladder perforation within the scrotum is a particularly severe and life-threatening complication of scrotal cystocele [1,3]. This complication, although exceedingly uncommon, introduces a new set of clinical challenges. In cases of bladder perforation, urine can extravasate into the surrounding scrotal and peritoneal tissues, leading to local inflammation, infection, and the formation of abscesses. The presence of urine within the scrotum can trigger an intense inflammatory response, promote fibrosis, and potentially damage adjacent anatomical structures, including the spermatic cord and testes. If not promptly recognized and treated, this can escalate to more severe complications, including urosepsis, a life-threatening systemic infection [2].

Diagnosis of scrotal cystocele, particularly when complicated by intraperitoneal or intrascrotal bladder perforation, requires a high degree of clinical suspicion. This is especially true for elderly patients with a history of recurrent inguinal hernias or those presenting with atypical symptoms. In many cases, patients may initially present with nonspecific symptoms, which can delay the diagnosis and complicate the clinical course. Imaging studies are crucial in the diagnostic process. Ultrasonography is often used as an initial modality to evaluate the presence of a herniated bladder within the scrotum and can identify associated complications such as bladder wall rupture. However, CT scanning provides a more detailed assessment and is considered the gold standard for visualizing the extent of bladder herniation and any associated perforation. CT scans offer cross-sectional images that allow for precise evaluation of the hernia’s size, location, and any signs of urinary extravasation into surrounding tissues [3].

Management of scrotal cystocele complicated by intrascrotal bladder perforation is inherently complex and requires a multidisciplinary approach involving urologists, general surgeons, and sometimes infectious disease specialists. Surgical intervention is the mainstay of treatment, and the goals of surgery are to repair the bladder perforation, reduce the hernia, and reinforce the weakened abdominal wall. In cases of bladder perforation, a partial cystectomy may be necessary to remove the damaged portion of the bladder. The repair of the hernia defect is typically performed using a mesh to provide long-term support to the weakened tissues [1,3]. The decision to use synthetic or biological mesh may depend on the presence of infection or contamination at the surgical site. In some cases, urinary diversion (e.g., placement of a suprapubic catheter) may be required postoperatively to allow the bladder to heal. The timing of surgical intervention is crucial, as delayed treatment increases the risk of complications such as severe infection, abscess formation, and long-term fibrosis, which can impair urinary function. Early diagnosis and prompt surgical repair are key to preventing these complications and improving patient outcomes [2,3]. Additionally, postoperative management should focus on optimizing the patient’s overall health, addressing risk factors such as obesity, and managing comorbidities to reduce the risk of recurrence. The management of scrotal cystocele complicated by bladder perforation underscores the need for clinical vigilance, particularly in high-risk populations. While this condition is rare, its potential for severe morbidity and even mortality necessitates timely recognition and intervention. This case highlights the importance of comprehensive diagnostic workups, the role of advanced imaging, and the critical need for surgical expertise in managing these complex presentations [1,2].

## Case presentation

An 83-year-old male patient presented to the emergency unit with complaints of left inguinal pain, a large left inguinal mass, and fever. Despite his advanced age, the patient was in remarkably good overall condition. He had been managing prostatic enlargement with a transurethral catheter. Clinical examination revealed a left inguinoscrotal hernia with signs of incarceration, accompanied by fever, indicating a potential complication.

Laboratory investigations

A complete blood count and renal function tests were done for this patient, and the positive findings are presented in Table 1.

**Table 1:** Lab parameters

Laboratory parameter	Patient’s value	Normal reference range	Interpretation
Total leukocyte count	16,050 cells/cu.mm	4,000-11,000 cells/cu.mm	Elevated, indicating a significant inflammatory response
Serum hemoglobin	9.4 g/dL	13.8-17.2 g/dL (males)	Low, indicating mild anemia
Neutrophils	78.9% of total WBCs	40-70% of total WBCs	Elevated, suggesting ongoing infection or inflammation
Serum urea	35 mg/dL	7-20 mg/dL	Normal
Serum creatinine	0.6 mg/dL	0.7-1.3 mg/dL	Normal

Imaging findings

**Figure 1:**
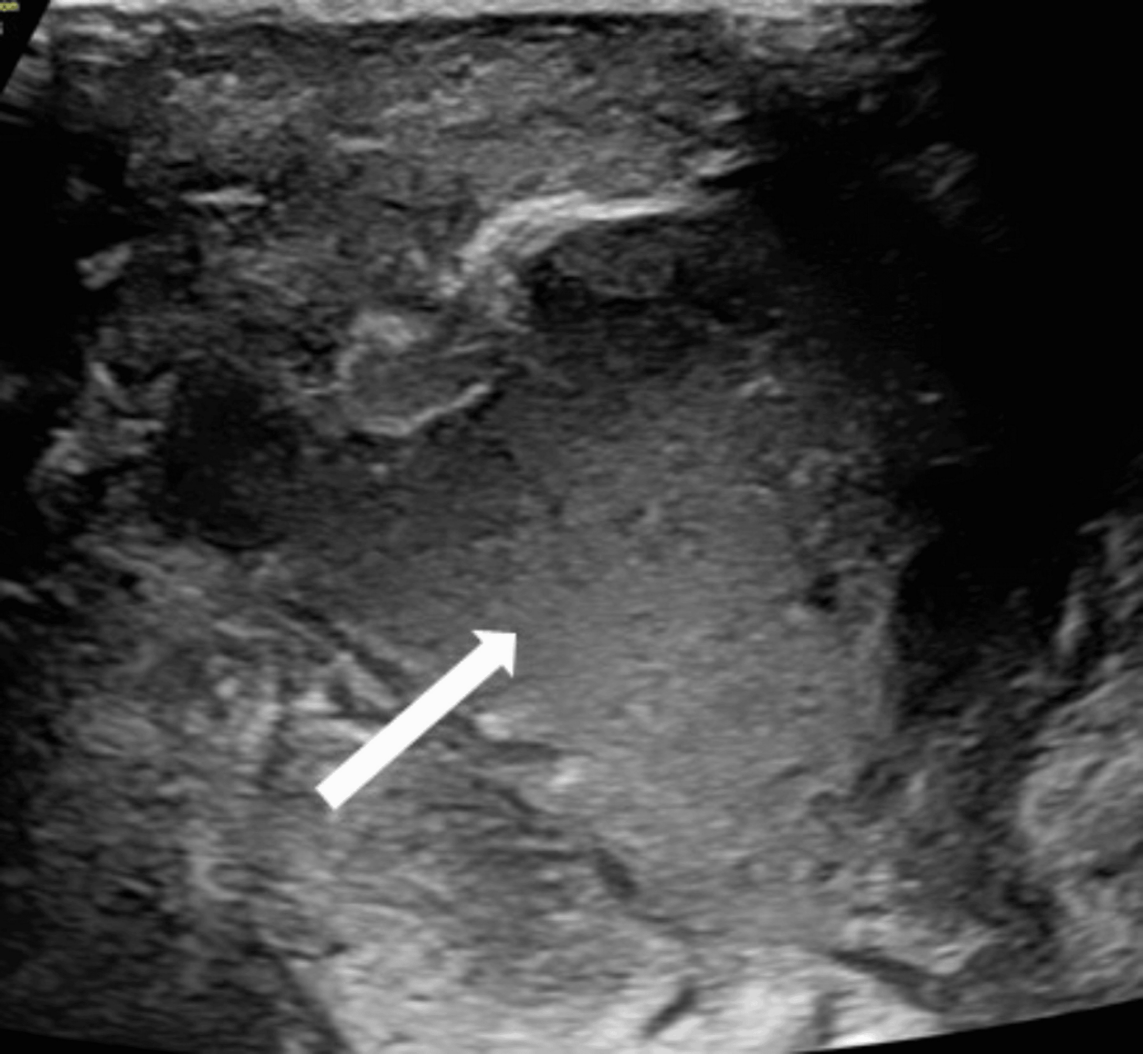
USG of the left scrotum shows hypoechoic collection with Brownian movements noted within the left scrotal sac (long white arrow) Brownian movements: random, erratic movement of small particles suspended in a fluid as they collide with the fast-moving molecules of the surrounding fluid USG, ultrasonogram

Ultrasound examination of the inguinoscrotal region revealed herniation of a portion of the urinary bladder into the left inguinal canal. Additionally, a hypoechoic collection with internal Brownian movements (random, erratic movement of small particles suspended in a fluid as they collide with the fast-moving molecules of the surrounding fluid). It was observed within the left scrotal sac, indicating the presence of fluid, likely infected or inflammatory in nature (Figure 1).

**Figure 2:**
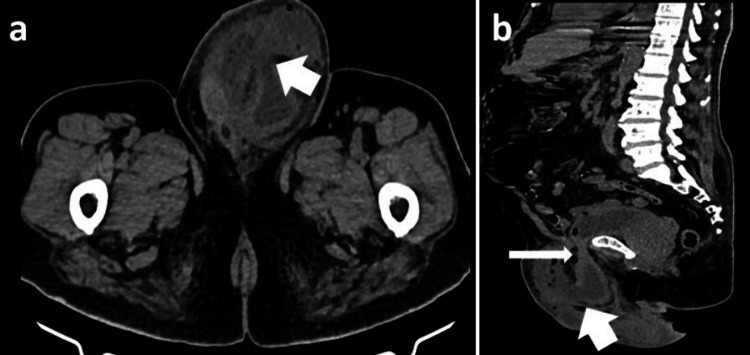
NCCT of abdomen and pelvis: (a) axial section showing a discontinuity in the wall of the herniated urinary bladder (short white arrow); (b) sagittal section showing herniation of the urinary bladder into the left inguinal canal (long white arrow) with a discontinuity in the wall of the herniated urinary bladder (short white arrow) NCCT, non-contrast CT

A non-contrast CT (NCCT) scan further delineated the left inguinal hernia, showing a portion of the urinary bladder herniated into the left scrotum. The scan also suggested a possible break in the wall of the herniated bladder, with fluid accumulation noted within the scrotal sac (Figure 2a, 2b).

**Figure 3:**
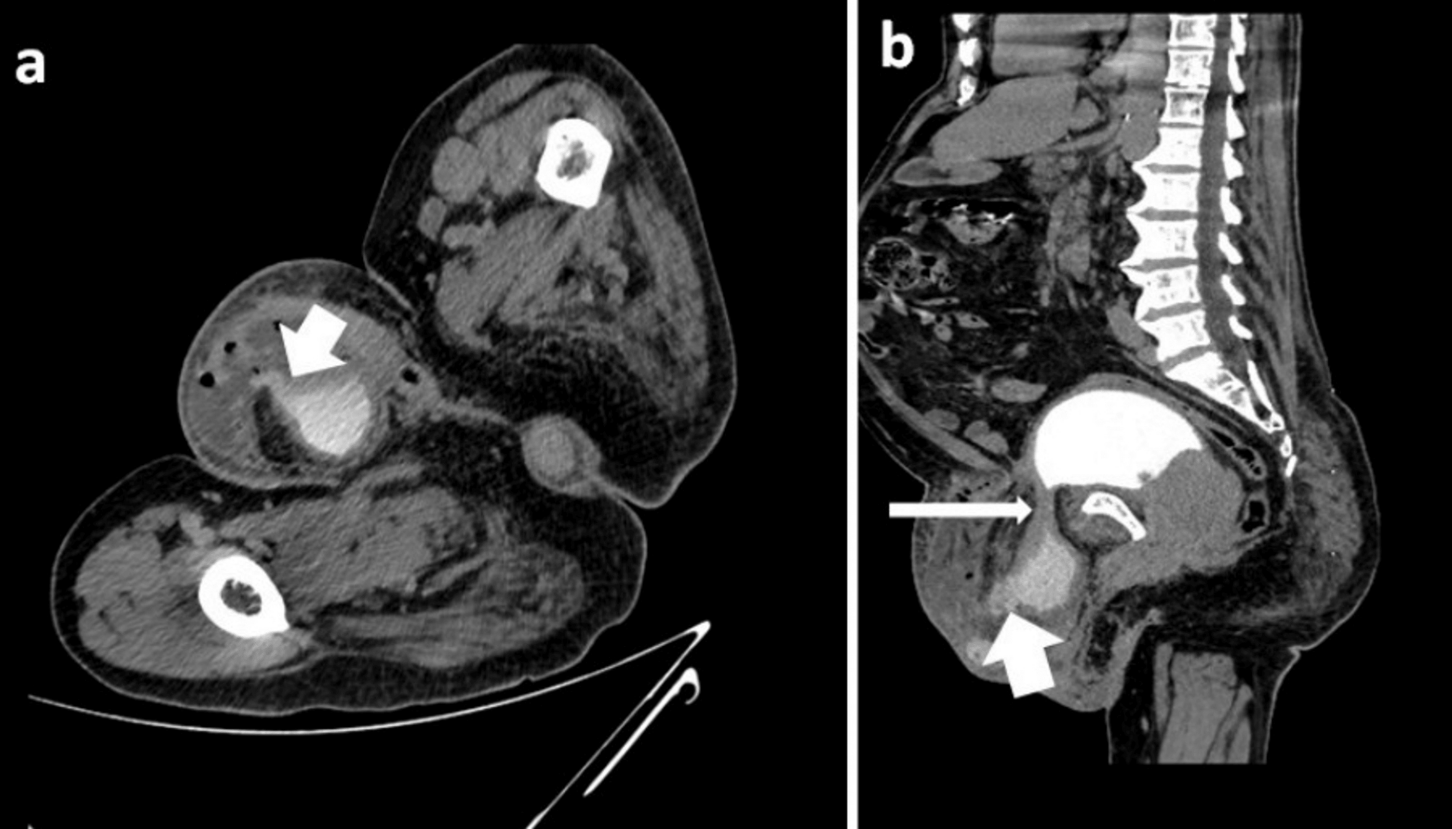
CECT abdomen and pelvis delayed phase: (a) axial section shows extravasation of contrast from the rent in the wall of herniated urinary bladder (short white arrow); (b) sagittal section shows herniation of urinary bladder into the left inguinal canal (long white arrow) and extravasation of contrast from the rent in the wall of herniated urinary bladder (short white arrow) CECT, contrast-enhanced CT

To obtain more detailed information, a contrast-enhanced CT (CECT) scan was performed, which confirmed the presence of a left inguinal hernia with a portion of the urinary bladder herniating into the left scrotum. The contrast-enhanced images revealed extravasation of contrast material through the bladder wall breaking into the left scrotal sac during the delayed phase, confirming the diagnosis of bladder perforation (Figure 3a, 3b).

Treatment

The patient underwent an urgent laparotomy, during which a partial cystectomy, debridement, left orchidectomy, and hernia repair were performed. A left inguinoscrotal incision was made, and the layers were carefully opened to access the herniated structures. The opening in the bladder diverticulum was extended to visualize the presence of the Foley catheter. A Pfannenstiel incision was then made, connecting with the previous incision to gain better access to the bladder. The bladder was identified, and a suprapubic catheter was placed to facilitate postoperative urinary drainage. The bladder diverticulum, which was found to be obstructed, was excised, and the bladder wall was sutured in two layers: an inner continuous layer and an outer interrupted layer.

**Figure 4:**
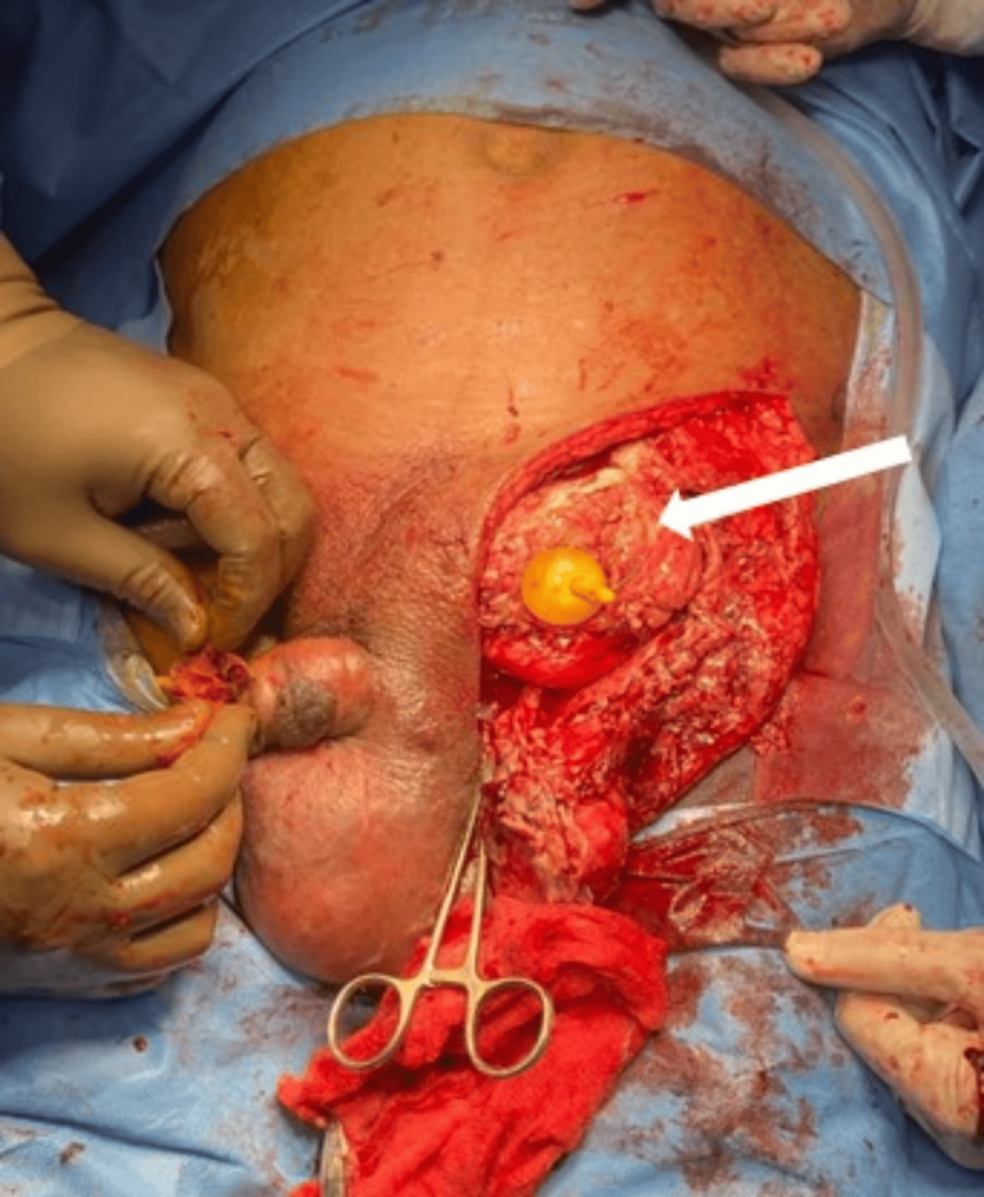
Intraoperative image showing exposed urinary bladder with Foley’s catheter in situ (long white arrow)

The hernial sac was reduced, with the bowel contents repositioned back into the abdominal cavity, and the hernia defect was closed using Vicryl sutures. To reinforce the repair, darning was performed using Prolene sutures. A left orchidectomy was conducted due to the compromised condition of the testicle. Hemostasis was meticulously achieved, and the surgical site was thoroughly irrigated with wound wash. A 14-size Romovac suction drain was placed to prevent postoperative fluid accumulation, and the wound was closed in layers. The excised specimen, which included a large obstructed diverticulum originating from the left lateral wall of the bladder, measuring 10 cm by 8 cm, was sent for histopathological examination. The diverticulum extended into the scrotum and had a perforation measuring 2 cm by 1 cm in its anterior wall. Approximately 50-60 cc of pus was aspirated from the scrotum, and the testis was found to be anatomically separate from the diverticulum (Figure 4).

**Figure 5:**
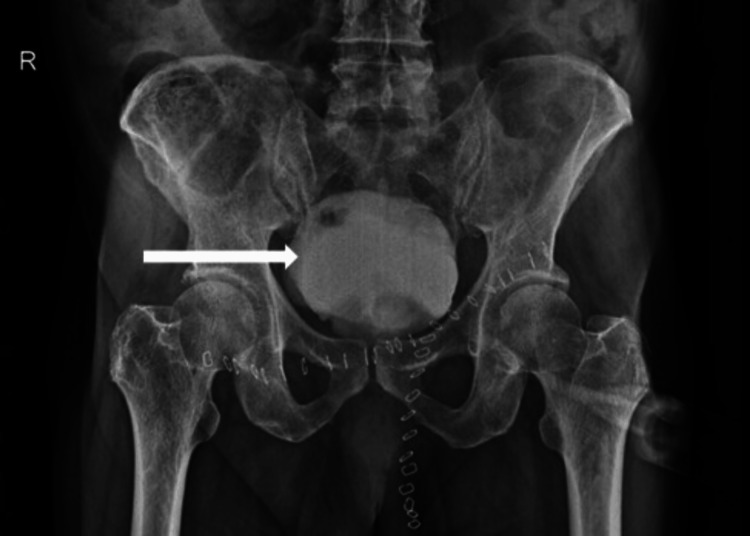
Postoperative X-ray cystogram shows contrast filling in the bladder with no evidence of any contrast leak (long white arrow)

A postoperative X-ray cystogram was performed, which demonstrated no leakage of contrast material from the bladder, confirming the integrity of the bladder repair (Figure 5).

The patient’s postoperative course was uneventful, and he was subsequently discharged in stable condition. The successful outcome in this case underscores the importance of prompt surgical intervention and meticulous postoperative care in managing such complex urological emergencies.

## Discussion

The herniation of the urinary bladder into the scrotal sac, particularly with subsequent intrascrotal perforation, presents a unique and complex clinical challenge. This rare condition, accounting for less than 4% of inguinal hernias, is predominantly observed in elderly, obese males [3]. The pathogenesis involves an acquired direct inguinal hernia where the bladder is pulled into the hernia sac, often accompanied by a sheath of the peritoneum. Several factors contribute to the development of bladder herniation, including bladder outlet obstruction, sliding direct inguinal hernias, obesity, and weakened pelvic musculature [4].

Clinically, bladder herniations are often asymptomatic, particularly when small [5]. However, larger herniations can lead to significant symptoms, such as a two-stage urination process, where initial voiding is spontaneous, followed by a second stage facilitated by manual compression of the scrotal mass [4,5]. This symptomatology, along with lower urinary tract infections, is characteristic of patients with significant bladder herniation [6].

The presence of bladder perforation within the scrotum adds a layer of complexity, often presenting with acute symptoms such as scrotal pain, swelling, and signs of systemic infection [3,6]. The differential diagnoses that can be considered for this condition include infected hydrocele and scrotal abscess. Diagnostic imaging plays a crucial role in the identification and management of this condition. Voiding cystography remains the gold standard for diagnosing bladder herniation. However, CT with contrast provides comprehensive information, particularly in identifying concurrent complications such as hydronephrosis or bowel involvement [7].

The case presented highlights the importance of utilizing multiple imaging modalities, including ultrasound, NCCT, and CECT, to confirm the diagnosis and assess the extent of the pathology. The management of bladder herniation with perforation is surgical, necessitating prompt intervention to prevent severe complications. In the reported case, the surgical approach involved laparotomy, partial cystectomy, debridement, left orchidectomy, and hernia repair [8]. This comprehensive surgical management underscores the need for a multidisciplinary approach involving urologists and general surgeons.

The use of a tension-free hernioplasty with a mesh is generally recommended for inguinoscrotal hernias; however, in the presence of acute inflammatory changes, as in this case, alternative surgical techniques must be employed. Postoperative care is equally critical in ensuring favorable outcomes. The successful recovery and discharge of the patient in the case report highlight the importance of meticulous surgical technique and careful postoperative monitoring [9]. Follow-up imaging, such as a cystogram, is essential to confirm the integrity of the bladder repair and ensure the absence of urinary leakage.

In conclusion, bladder herniation into the scrotal sac with intrascrotal perforation is a rare but serious condition that requires a high index of suspicion for timely diagnosis [7,8]. Multimodal imaging is vital for accurate assessment, and prompt surgical intervention is crucial to mitigate risks and achieve optimal patient outcomes [10].

Literature review

A comprehensive review of the existing literature on this topic is summarized in Table 2.

**Table 2:** Literature review

Author(s)	Year	Title	Findings
Levine [1]	1951	Scrotal cystocele: report of a case (Oxford Academic)	First description of bladder herniation into the scrotum
Zotani et al. [2]	2024	A case of indirect inguinal bladder hernia treated with laparoscopic transabdominal preperitoneal repair with high peritoneal incisional approach	Case report describing the successful treatment of an indirect inguinal bladder hernia using a laparoscopic approach
Elkbuli et al. [3]	2019	Inguinal bladder hernia: a case report and literature review	Inguinal bladder hernia with diagnostic and therapeutic challenges, emphasizing imaging and surgical strategies
Wang et al. [4]	2018	Large sliding inguino-scrotal hernia of bladder: a case report and literature review	Case report and literature review on surgical techniques for managing large bladder hernias
Aguado et al. [5]	2001	Giant inguino-scrotal bladder hernia: report of a case	Case report of giant bladder hernia managed with modified Lichtenstein technique
Kraft et al. [6]	2008	Inguinoscrotal bladder hernias: report of a series and review of the literature	Review of inguinoscrotal bladder hernias, including surgical management options and outcomes
Karanikas et al. [7]	2020	Urinary bladder-containing incarcerated inguinoscrotal hernia: a case report	Discussed clinical presentations and surgical outcomes of inguinoscrotal bladder hernias
Giglio et al. [8]	2001	Scrotal extraperitoneal hernia of the ureter: case report and literature review	Detailed case report and review of extraperitoneal hernias involving the bladder and ureter
Lee et al. [9]	2014	Inguinoscrotal herniation of bladder: case series and literature review	Series of cases exploring the complexities of diagnosing and treating bladder herniation into the scrotum
Shakil et.al [10]	2020	Inguinal hernias: diagnosis and management	Comprehensive overview of inguinal hernia diagnosis and management strategies

## Conclusions

This case highlights the successful management of a rare and complex urological emergency involving bladder herniation into the scrotal sac with associated bladder perforation. The prompt recognition of the condition, guided by detailed imaging and a comprehensive surgical approach, was crucial in achieving a favorable outcome. The surgical intervention, which included partial cystectomy, debridement, hernia repair, and left orchidectomy, effectively addressed the complications, while meticulous postoperative care ensured the patient’s recovery. This case underscores the importance of a multidisciplinary approach and the need for heightened clinical suspicion in similar presentations to improve patient outcomes in such challenging cases.
